# Impact of socioeconomic status and medical conditions on health and healthcare utilization among aging Ghanaians

**DOI:** 10.1186/s12889-015-1603-y

**Published:** 2015-03-20

**Authors:** Bashiru II Saeed, Zhao Xicang, Alfred Edwin Yawson, Samuel Blay Nguah, Nicholas NN Nsowah-Nuamah

**Affiliations:** School of Finance and Economics, Jiangsu University, Zhenjiang, China; Mathematics and Statistics Department, Kumasi Polytechnic, Kumasi, Ghana; Department of Community Health, College of Health Sciences, University of Ghana, Korle-Bu Accra, Ghana; Paediatric Department, Komfo Anokye Teaching Hospital, Kumasi, Ghana; Kumasi Polytechnic, Kumasi, Ghana

**Keywords:** Subjective health, Socioeconomic status, Healthcare utilization, Functioning assessment, Aged Ghanaians

## Abstract

**Background:**

This study attempts to examine the impact of socioeconomic and medical conditions in health and healthcare utilization among older adults in Ghana. Five separate models with varying input variables were estimated for each response variable.

**Methods:**

Data (Wave 1 data) were drawn from the World Health Organization Global Ageing and Adult Health (SAGE) conducted during 2007–2008 and included a total of 4770 respondents aged 50+ and 803 aged 18–49 in Ghana. Ordered logits was estimated for self-rated health, and binary logits for functional limitation and healthcare utilization.

**Results:**

Our results show that the study provides enough grounds for further research on the interplay between socioeconomic and medical conditions on one hand and the health of the aged on the other. Controlling for socioeconomic status substantially contributes significantly to utilization.

Also, aged women experience worse health than men, as shown by functioning assessment, self-rated health, chronic conditions and functional limitations. Women have higher rates of healthcare utilization, as shown by significantly higher rates of hospitalization and outpatient encounters.

**Conclusion:**

Expansion of the national health insurance scheme to cover the entire older population- for those in both formal and informal employments- is likely to garner increased access and improved health states for the older population.

## Background

Global population of the aged is on the rise. Statistics of the past two decades present a surge in aged population across countries as a result of improved indices in life expectancy and slowed mortality globally [1]. In addition to these, improved health and economic growth in many developing countries have provided a favourable condition for this outcome.

The aged population is a vulnerable community due to high susceptibility to plaguing life conditions such as morbidity and mortality [2,3]. There is therefore widespread discourse on the need to improve their living conditions; however this has not garnered needed priority in the arena of development policy in many developing countries. The aged population is widely left to the care of traditional institutions like the family as policy and institutional framework to manage later year’s needs are not concretely defined.

Previous studies on the aged have established that older women are universally more exposed to socioeconomic vulnerabilities which affect health and economic outcomes [4]. Evidence from literature also points to higher longevity among women than men thus increasing the proportion of women in old age [5]. In spite of higher longevity, studies from developed countries indicate a higher frequency of morbidity among women which leads to increased utilization of healthcare services [6,7].The situation is quite different from developing countries although empirical studies on the aged are quite difficult to come by. A study by Roy and Chaudri [8] in India argued that there is statistically significant differential in socio-economic and cultural experiences of older men and women. Older women have lower levels of literacy, property ownership, economic independence and social integration than men. This is also worsened by a high likelihood of widowhood and dependent living arrangements. All these factors negatively influence health status and healthcare utilization among older women including lower rates of hospitalization and outpatient encounters.

Thoa *et al*. [9] also argued based on a study of rural Vietnam that healthcare utilization changes in direct relationship to changes in economic conditions of households. In effect, factors that contribute to weakening household economic growth have the likelihood to affect healthcare utilization and broadly heath status in older ages.

About sixty-four per cent of aged population resides in developing countries and this is projected to increase to 80 per cent by 2050 [9,10]. In tandem, Africa’s older population is expected to increase almost fourfold in that period from 36.6 million to 141 million [11]. Average life expectancy at birth in Sub Sahara Africa is projected to increase from about 45 years at present, to 63 years by 2050, resulting in an increase in the population of the aged in the region.

With respect to Ghana, the national population according to the Population and Housing Census [12] stands at 24.7 million with dependent population (persons under 15 and over 65 years) constituting 43.1%. The aged population, which by definition include persons 60 years and above [13] is 6.5% of the population. The dependency ratio is expected to increase significantly given population policy objectives of reducing child mortality and increasing life expectancy [13]. It has also been established that life expectancy has seen upward growth from the time of independence, from 42 years in 1960 to 58 years in 2000, and it is expected to increase to 65 years by 2020 and 70 years by 2050. To improve access to health care by the citizenry, Ghana introduced a National Health Insurance Scheme (NHIS) to replace an existing pay-as-you access (cash and carry) payment system by an act of parliament in 2003 [14]. This policy has affected virtually all aspects of health system such as improved access, workload, health infrastructure and care for vulnerable groups including children, aged and pregnant women [15,16].

Although research on ageing and adult health has been conducted, or is ongoing in several South Sahara African countries including Ghana, these studies have typically been small and piecemeal [17,18]. This analysis is based on SAGE Wave 1, a multi-country longitudinal study by the World Health Organization to provide nationally representative data (including the health state, health care utilization and health system responsiveness) on older adults in six low-to-middle income countries including Ghana.

This paper seeks to explain the relationships between socioeconomic, socio-demographic factors, functioning assessment and chronic conditions that influence health and healthcare utilization among the older population in Ghana. There is generally paucity of literature on the aged in areas of health and healthcare utilization in developing countries [19] which feeds directly into the low level of concrete evidenced-based policies to advance the cause of the aged in these settings. The paper therefore intends to contribute to providing nationally representative analysis of the health care utilization patterns among the older population of Ghana to augment national health and social policy.

## Methods

### Study design

This was a longitudinal study of older adults with nationally representative samples of persons aged 50+ years in Ghana with a smaller sample of adults aged 18–49 years. Instruments are compatible with other large high-income country longitudinal ageing studies. In this article, 4770 older persons had been considered. ‘SAGE has been approved by the World Health Organization’s Ethical Review Board. Additionally, the study in Ghana was approved by the Ethical and Protocol Review Committee of the University of Ghana Medical School, Korle-Bu, Accra. Informed consent has been obtained from all study participants’.

### Study sites

SAGE Wave 1 was conducted in all the ten regions of Ghana in 2007/2008.

SAGE also includes two longitudinal sub-studies to develop further hypotheses, assess the effects of interventions and study specific populations, based on collaborations with existing demographic and health surveillance systems field sites and local community studies. The SAGE collaboration with the International Network for the Demographic Evaluation of Populations and Their Health in developing countries (INDEPTH; www.indepth-network. org) involves collaboration with eight field sites in eight countries.7–9 One round of data collection took place in 2007, with a second round planned to start in 2013. Further sub-studies include the validation of self-reported physical activity against accelerometry measures in older adults (paa2012.princeton.edu/papers/122841), the assessment of happiness through different versions of the Day Reconstruction Method, 12, 13 and the validation of self-reported chronic conditions through confirmatory medical tests.

### Study population

SAGE is a longitudinal study with nationally representative samples of persons aged 50+ years in Ghana, with comparison samples of younger adults aged 18–49 years in Ghana. The face-to-face interview was conducted in Ghana (2007–08). Multistage cluster sampling strategies were used where households were classified into one of two mutually exclusive categories:

1. All persons aged 50 years and older were selected from households classified as ‘50+ households’; and
2. One person aged 18–49 years was selected from a household classified as an ‘18–49 household’ (i.e. a household without a person ≥ 50 years).

Household enumerations were carried out in the final sampling units. One household questionnaire was completed per household where a household informant and individual respondent need not be the same individual. One individual was selected from 18–49 households, whereas for 50+ households all individuals aged 50+ were invited to complete the individual interview. For selected individuals who were unable to respond to the interview questions, a member of the household (proxy respondent) was made to assist. Household-level analysis weights and person-level analysis weights were calculated for each country, which included sample selection and a post-stratification factor. Post stratification correction techniques used the most recent population estimates provided by the Ghana Statistical Service. The pooled Wave 1 national total for individual respondents included 4770 respondents aged 50+ and 803 aged 18–49.

### Study procedure

A standardized survey instrument, set of methods, interviewer training and translation protocols are used in all SAGE countries.

International standards were used to harmonize education levels and occupations. Details on subject selection and instruments used for the SAGE Wave 1 survey across the six countries have been provided by Kowal *et al.* [17] and that in Ghana specifically by Biritwum et al. [18].

### Statistical methods

#### Measures of variables

Response variables include two measures of health status and a measure of healthcare utilization. Self-rated health status indicates how Ghanaians rated their health state by using five-point scale ranging from “very good” to “very bad” coded as categorical variable that ranges from 1 equals “very good” to 5 equals “very bad”. Objective health state is measured on the basis of the Ghanaians response to a question (Q2002) regarding the presence of functional limitations (mild to extreme = 1) denoted as present and (none = 0) denoted as absent respectively. Healthcare utilization is measured using self-reports of hospitalization (outpatient) during a one year reference period preceding the survey.

#### Socio-demographic and socio-economic measures

Predisposing measures were age (age 18–49 (young adults), 50–59 (adults) and 60 yrs and above (older adults)), gender and marital status (currently married, never married, cohabitating, separated/divorced and widowed), ethnicity, and religion. These sociodemographic variables were chosen because of their influence on chronic diseases, health status and health care utilization respectively.

Enabling measures were assessed in terms of education, job employment, social class, insurance status, area of residence and income. Education was recorded as *college/university completed, high school completed, secondary school completed, primary school completed, less than primary school completed and no formal education*. Job employment was categorized into four groups: public, private, self-employed and informal employment. Public sector includes employees of state, or municipal governments and their agencies, parastatal enterprises, and semi-autonomous institutions such as social security institutions that are owned by the government or institutions like religious schools if the staff are paid by the government. Private sector includes any employees not working for the government and not self-employed. Self-employed includes those who earn their livelihood directly from their own trade or business rather than as an employee of another. Informal employment could mean employment in the informal economy or informal employment. Informal economy refers to the general market income category wherein certain types of income and the means of their generation are “unregulated by the institutions of society, in a legal and social environment in which similar activities are regulated”. Jobs in the informal economy are characteristically without benefits such as health insurance, sick leave, paid vacations or pensions. Insurance status was recorded as respondents who have health insurance coverage. Social class status was recorded as *completely, mostly, moderately, a little and not at all*. Income level was divided into five categories: Income Quintile; *Q1 (lowest) through Q5 (highest)*. Wealth or income Quintiles were derived from the household ownership of durable goods, dwelling characteristics (type of floors, wells and cooking stove), and access to services (improved water, sanitation and cooking fuel) for a total of 21 assets. A two-step random effects probit model was used to generate the Quintiles (Kowal, *et al.* [17]). Again, these socioeconomic variables were chosen because of their influence on chronic diseases, health status and health care utilization respectively.

Functioning assessment are measured on the basis of difficulties Ghanaians have had due to illnesses in the past 30 days. They include cognition, impaired vision, mobility, sleep and energy and depression and were carefully included because of their influence on health status and health care utilization respectively Chronic illnesses are measured on the basis of some health problems Ghanaians may have experienced including angina, hypertension, stroke, diabetes, arthritis, visual impairment and obesity. These were considered because of their influence on health care utilization.

### Statistical analysis

Maximum likelihood multivariate regression analysis was applied in the analysis to examine Ghanaians differences in self-rated health, functional limitation and healthcare utilization in both different samples for males and females. Ordered logit model was estimated for self-rated health, and binary logit model for functional limitation and healthcare utilization.

Five separate models with varying input variables were estimated for each response variable.

The first model controls for predisposing factors for each health estimate, and then successively introduce another set of input variables in order of chronic illnesses, functioning assessment and enabling factors.

Stata SE (version 12.1) was used for analysis.

### Ethical considerations

‘SAGE has been approved by the World Health Organization’s Ethical Review Board. Additionally, the study in Ghana was approved by the Ethical and Protocol Review Committee of the University of Ghana Medical School, Korle-Bu, Accra. Informed consent has been obtained from all study participants’.

## Results

Table 1 shows the differences exhibited in enabling and predisposing factors among aged male and female Ghanaians. Females show significantly higher levels of illiteracy, urban dwelling and self-employment than men. Women had low level of education and higher rates of single parenthood, compared with aged men.

**Table 1:** **Weighted means and percentages of selected demographic, socioeconomic and health variables by gender**

	**Male**	**Female**	**All**	**Male–female**	
				**Difference**	**P value**
Mean Age	74.42	73.88	74.15	0.54	0.1686
Married	80.22	21.66	50.94	58.56	0.0000
Akan Tribe	44.67	52.32	48.50	−7.65	0.0052
Illiterate	54.45	81.70	68.08	−27.25	0.0000
Urban residents	39.90	40.50	40.20	−0.6	0.8247
Self employed	72.57	86.06	79.32	−13.49	0.0000
Hypertension	57.96	63.08	60.52	−5.12	0.0498
Diabetes	3.36	4.67	4.02	−1.31	0.1805
Arthritis	15.31	20.54	17.93	−5.23	0.0216
Obesity	6.20	8.59	7.40	−2.39	0.0461
Visual impairment	34.89	38.52	36.71	−3.63	0.1325
Difficulty Moving Around	55.78	65.21	60.50	−9.43	0.0001
Resting issues	62.58	71.79	67.19	−9.21	0.0001
Sleeping Issues	62.06	71.06	66.56	−9.00	0.0001
Depressed Feeling	54.24	61.40	57.82	−7.16	0.0017
Hospitalized, last 12 months	66.98	71.72	69.35	2.37	0.0588

The weighted means and percentages for the sampled health related indicators also shown (Table 1) demonstrate that aged women experience worse health than men, indicated by functioning assessment, self-rated health, chronic conditions and functional limitations.

Overall, women had higher rates of healthcare utilization, indicated by the significantly higher rates of hospitalization and outpatient encounters.

Tables 2, 3 and 4 respectively show results from the regression output, conducted on the pooled sample. In these, different measures of health and healthcare were regressed, (predisposing factors, experiences of medical conditions and enabling factors as input variables were controlled for in a cumulative order in the respective model specifications).

**Table 2:** **Coefficients from the ordered logit regression on self-assessed health**

**Variables**					
	**Model 1**	**Model 2**	**Model 3**	**Model 4**	**Model 5**
Age	.05467 [.1033]	.0334 [.1008]	.0048[.1002]	-.0038 [.1029]	.0008 [.0998]
Age^2^	-.0001 [.0007]	.0000 [.0006]	.0002 [.0006]	.0002 [.0007]	.0002 [.0006]
Married	-.2567** [.1015]	-.2437** [.1028]	-.1288 [.1101]	-.0857 [.1095]	-.0689 [.1071]
Akan	-.0152 [.1076]	-.0068 [.1076]	.0381 [.1086]	.0587 [.1073]	.1230 [.1121]
Hypertension		.0182 [.1036]	-.0604 [.1074]	-.0376 [.1085]	-.0105 [.1092]
Diabetics		.6389*** [.1969]	.5718*** [.2166]	.6017*** [.2189]	.6433*** [.2181]
Angina		.8234*** [.2159]	.8735*** [.2294]	.8875*** [.2298]	.8892*** [.2252]
Arthritics		.1335 [.1329]	-.0655 [.1342]	-.0617 [.1361]	-.1165[.1406]
Stroke		.3940 *[.2360]	.1305 [.2442]	.1596 [.2516]	.1047 [.2609]
Visual impairment		.2690 ** [.1031]	.1470 [.1064]	.1337 [.1061]	.1225 [.1056]
Obese		-.2051 [.1661]	-.0612[.1760]	.0076 [.1894]	.0225 [.1904]
Memory			.7115*** [.1246]	.6895*** [.1253]	.6874*** [.1258]
Visual diff			.1274 [.1156]	.1283 [.1170]	.1191 [.1183]
Moving			.9464*** [.1120]	.9484*** [.1114]	.9456 *** [.1104]
Sleep diff			.0877 [.1237]	.07335 [.1238]	.0622 [.1227]
Employment status				.1066 [.1460]	.0912[.1483]
Low income				-.1913 [.1556]	-.1587 [.1607]
High income				-.2230 [.1681]	-.1544 [.1713]
Higher income				-.1889 [.1583]	-.0846[.1664]
Highest income				-.3836** [.1837]	-.2207 [.2045]
Urban/rural				-.0038 [.1296]	.0082 [.1271]
Illiteracy					.0465 [.1267]
Observations	2142	2142	2142	2142	2142
F-statistic	10.24	8.15	14.74	11.82	11.47
D.F	213	213	213	213	213
Prob > F	0.0000	0.0000	0.0000	0.0000	0.0000

Note: ***, ** and * indicates1%, 5% and 10% significance level respectively.

**Table 3:** **Coefficients from the logit regression on probability of functional limitation**

**Variables**					
	**Model 1**	**Model 2**	**Model 3**	**Model 4**	**Model 5**
Age	.1327[.1060]	.0670 [.1020]	.0301[.1038]	.0398 [.1031]	.0358 [.0991]
Age^2^	-.0006[.0007]	-.0002 [.0007]	.0000 [.0007]	-.0000 [.0007]	-.0000 [.0006]
Married	-.3863*** [.0909]	-.3464 *** [.0898]	-.2767 *** [.0879]	-.2611*** [.0919]	-.2418 *** [.0924]
Akan	-.0179 [.1274]	-.0520 [.1224]	-.0272 [.1165]	-.0477 [.1186]	.0172 [.1160]
Hypertension		.1995*** [.0927]	.1838** [.0927]	.1733* [.0931]	.1992** [.0925]
Diabetics		.1370[.2256]	.0667 [.2229]	.0811[.2236]	.1150 [.2182]
Angina		.05157 [.3008]	.0878 [.3007]	.0854 [.2863]	.1048 [.2826]
Arthritis		.6874***[.1251]	.5315***[.1245]	.5385***[.1230]	.4836***[.1242]
Stroke		1.1707 *** [.2434]	1.0579*** [.2300]	1.0555*** [.2341]	1.0228*** [.2368]
Visual Impairment		.5355*** [.0991]	.4805*** [.0957]	.4770*** [.0967]	.4579 *** [.0967]
Obese		.0903 [.1923]	.3608* [.1973]	.3557* [.1933]	.3691** [.18967]
Memory			.4933*** [.1189]	.4958*** [.1187]	.4939*** [.1213]
Visual diff			.4573*** [.1244]	.4490*** [.1249]	.4489*** [.1242]
Sleep diff			1.0132*** [.1236]	1.003*** [.1224]	.9911*** [.1226]
Employment status				.0697 [.1392]	.0484 [.1371]
Low income				.2959** [.1504]	.3334** [.1486]
High income				.2810* [.1653]	.3529** [.1675]
Higher income				.0505 [.1593]	.1594 [.1629]
Highest income				-.0007 [.1697]	.1600 [.1769]
Urban/rural				-.1056 [.1313]	-.1042 [.1305]
Illiteracy					.0636 [.1112]
Social class					-.3813*** [.1132]
Observation	2142	2142	2142	2142	2142
F-statistic	17.87	15.55	18.56	14.79	13.50
D.F	213	213	213	213	213
Prob > F	0.0000	0.0000	0.0000	0.0000	0.0000

Note: ***, ** and *indicates1%, 5% and 10% significance level respectively.

Firstly, ageing Ghanaians experience significantly poorer self-rated health after controlling for married Ghanaians as a predisposing factor (model 1). This is because many are on low incomes and possibly living in rural settings where public utilities are difficult to access and hence worse health state. After controlling for chronic illness, the effect of diabetes, angina and visual impairment, statistically significantly influence on self-rated health (model 2) which implies that the differential exposure of ageing Ghanaians to these chronic conditions cannot explicitly explain gender differences. Interestingly, as we continue to control for functioning assessment.

**Table 4:** **Coefficients from the logit regression on probability of hospitalization**

**Variables**					
	**Model 1**	**Model 2**	**Model 3**	**Model 4**	**Model 5**
Age	-.0024 [.1036]	.0076 [.1026]	.0082 [.1038]	.0235 [.1032]	.0334 [.1067]
Age^2^	-.0001 [.0007]	.0001 [.0007]	.0000 [.0007]	-.0001 [.0007]	-.0001 [.0007]
Married	-.0298 [.1063]	-.0246 [.1086]	-.0115 [.1084]	-.1215 [.1100]	-.1554[.1107]
Akan	.3199 [.1262]	.32018*** [.1289]	.3335*** [.1280]	.2413 *[.1278]	.1592[.1303]
Hypertension		.3109*** [.1139]	.3137*** [.1143]	.2382** [.1173]	.2143* [.1188]
Diabetics		1.3139*** [.3440]	1.2933 *** [.3426]	1.1914 *** [.3394 ]	1.1543*** [.3428]
Angina		.9861*** [.3683]	.9833 *** [.3657]	.9237*** [.3638]	.9245 *** [.3629]
Arthritis		-.1213 [.1569]	-.0769 [.1586]	-.0783 [.1586 ]	-.0227 [.1552]
Stroke		.0988 [.3722]	.0806 [.3688]	.0006 [.3599 ]	.0323 [.3503]
Visual impairment		-.1001[.1086]	-.0943 [.1097]	-.0762 [.1118 ]	-.0603[.1116]
Obese		-.3551 [.2323]	-.3350 [.2375]	-.5360** [.2434]	-.5704**[.2433]
Memory			.2678** [.1374]	.2914** [.1399 ]	.3124 **[.1401]
Visual diff			-.0755 [.1352]	-.0908** [.1374]	-.0794[.1411]
Sleep diff			-.1424 [.1269]	-.1076 [.1279 ]	-.0910[.1296 ]
Employment Status				.1586 [.1667 ]	.1933 [.1649 ]
Low income				.3735** [.1678 ]	.3324**[.1713 ]
High income				.7035 *** [.1597]	.6267*** [.1634]
Higher income				.7893*** [.1905]	.6800 *** [.1931]
Highest income				.8283 *** [.2194]	.6404*** [.2326 ]
Urban/rural				-.0975 [.1401]	-.0981 [.1420]
Illiteracy					-.1802 [.1408]
Social class					.3906*** [.1316]
Observations	2142	2142	2142	2142	2142
F-statistic	2.56	4.04	3.83	4.30	5.03
D.F	213	213	213	213	213
Prob > F	0.0394	0.0000	0.0000	0.0000	0.0000

Note: ***, ** and * indicates1%, 5% and 10% significance level respectively.

After controlling for chronic illness, the effect of diabetes, angina and visual impairment, had statistically significant influence on self-rated health (model 2). Interestingly, when functioning assessment was controlled for, difficulties in movement due to illness and memory problems remained highly influential on self-assessed health as demonstrated in model 3. In model 4, after controlling for the enabling factors, urban settings, income quintile 5 (highest income group) and subjective well-being had less impact on the self-assessed health(Table 2), i.e. indicating that financially sound Ghanaians have better health state and are able to care for themselves.

In Table 3, upon the inclusion of education and social class to the model, functional limitation was greatly influenced by social class as an independent factor. Upon inclusion of chronic conditions and functioning assessment into the model, hypertension, arthritis, stroke and visual impairment contributed massively to the functional limitation.

Those in the Urban settings who earn income and influential in society have greater chance of receiving healthcare (probably because financially sound Ghanaians might have improved financial access to care).

Aged Ghanaians who were married reported worse health status and functional limitation (Table 3) than their non-married counterparts, whereas Akans report higher hospitalization (Table 4) than other tribes. However, marital status does not significantly impact outpatient hospitalization. Again, being an Akan does not significantly impact health status and functional limitation. Aged respondents who earn income and in high social class reported lower functional health and higher rates of hospitalization. Higher prevalence of functioning assessment and chronic conditions significantly increases the likelihood of hospitalization among aged Ghanaians (Tables 3 and 4) and also increases both subjective and functional health (Table 2). In total, enabling factors are among the greatest indicators of health and healthcare utilization among aged Ghanaians. Higher enabling factors have substantial and significant effects on outpatient care. Income and social class have strong beneficial impacts on functional limitation and outpatient care.

However, urban dwelling and education were neither associated with health nor healthcare utilization.

## Discussion

This study seeks to provide nationally representative baseline information on the aged population by examining disparities in health and healthcare utilization among aged Ghanaians. The analysis demonstrates huge differences in indicators of health state and healthcare utilization among aged Ghanaians.

The findings indicate income and social class have strong beneficial impacts on functional limitation and outpatient care. However, urban dwelling and education were neither associated with health nor healthcare utilization. This confirms Thoa et al. [3] who argued based on the use of longitudinal analysis on a study of rural Vietnam that healthcare utilization changes in direct relationship to changes in economic conditions of households. Again a similar study by Roy and Chaudri [8] conducted in India argued that there is statistically significant differential in socio-economic and cultural experiences of older men and women.

Our findings also showed the differences exhibited in enabling and predisposing factors among aged male and female Ghanaians. Females show significantly higher levels of illiteracy, urban dwelling and self-employment than men. This was agreed by Roy and Chaudri [8] who confirmed that older women have lower levels of literacy, property ownership, economic independence and social integration than men. The situation of the older woman is also worsened by a high likelihood of widowhood and dependent living arrangements. Previous studies have established that older women are universally more exposed to socioeconomic vulnerabilities which affect health and economic outcomes (United Nations, [10]). This finding conforms to our current analysis which demonstrates that aged women experience worse health than men, indicated by functioning assessment, self-rated health, chronic conditions and functional limitations. Women have higher rates of healthcare utilization, as shown by significantly higher rates of hospitalization and outpatient encounters. A lack of information relevant and specific to older persons’ physical, cognitive, social and economic well-being may contribute to their marginalization in policy arena (Kinsella & Phillips, [19]). The evidence provided by this analysis that enabling factors are among the greatest indicators of health and healthcare utilization among aged Ghanaians should contribute to national development policy.

In addition, the analysis demonstrated higher rates of healthcare utilization among the older population with poorer subjective health.

This suggests that aged Ghanaians with the worse health utilize more the limited health services available. In contrast to these, a study by Roy and Chaudri in 2008 [8] observed among aged Indians that are poorer subjective health impacts less on hospitalization. We have established in this study that controlling for socioeconomic status contributes substantially to utilization; possibly because financially sound Ghanaians might have increased financial access to healthcare. The older population in the higher socioeconomic group probably may be more aware and also may have increased health risks due to lack of exercise, being more sedentary, increased intake of high fat-laden diets and hence more need for outpatient care. Structured national health awareness programmes for the older population is imperative, Roy and Chaudri [8] provide counter explanation to the above that, the financially empowered and better-off older adults possibly have better health and need less inpatient care. Higher enabling factors has substantial and significant effects on healthcare utilization and in congruence with Roy and Chaudri [8], the effect of financial empowerment enhances health and well-being among both older men and women. In this analysis, older persons in the second and third income quintiles have challenges in overcoming functional limitation probably due to low savings and reduced access to health care.

Previous literature indicates that low-income and aged societies with no social fund to care for their growing older population, financial empowerment of older adults could be one way to improve their health outcomes, lower healthcare utilization and control long-term costs (Jerliu et al. [4]). In Ghana improving access to health care through removal of financial and other cost barriers through the national health insurance scheme (NHIS) is essential. The current operation of the NHIS which provides access to older persons above 60 years in the formal sector needs to be expanded to cover the entire older population- for those in both formal and informal employments (NHIS Annual report 2012 [14]).

The major limitation of the study is that, cross-sectional data from SAGE Wave 1 will largely not permit cause and effect examination. Enabling factors and medical conditions discussed in this analysis can affect poor health and at the same time could lower economic status and worsen medical conditions. The ongoing longitudinal study with SAGE wave 2 planned for 2013–2014 and wave 3 subsequently could serve as a remedy to this. In many survey data, subjective health is recorded using self-reports by point scale which might be influenced by the differences in way of reporting by respondents. This study attempted to resolve this by applying more health indicators and also by controlling for the distribution differences of functioning assessment and chronic illnesses.

## Conclusion

In conclusion, the current study provides enough grounds for further research on the interplay between socioeconomic and medical conditions on one hand and the health of the aged on the other. A structured and targeted national health awareness programme (taking cognizance of gender and socioeconomic differences in health care utilization) for the older population should be seen as essential development policy by the national government.

Expansion of the national health insurance scheme to cover the entire older population- for those in both formal and informal employments- is likely to garner increased access and improved health states for the older population.
